# The impact of headache and chronic musculoskeletal complaints on the risk of insomnia: longitudinal data from the Nord-Trøndelag health study

**DOI:** 10.1186/1129-2377-14-24

**Published:** 2013-03-12

**Authors:** Siv Steinsmo Ødegård, Trond Sand, Morten Engstrøm, John-Anker Zwart, Knut Hagen

**Affiliations:** 1 Department of Neuroscience; Faculty of medicine, Norwegian University of Science and Technology, MTFS, Trondheim N-7489, Norway; 2 Norwegian National Headache Centre, Section of Neurology, St. Olavs Hospital, Trondheim, N-7006, Norway; 3 FORMI and Department of Neurology, Oslo University Hospital and Faculty of Medicine, University of Oslo, Oslo, N-0450, Norway

**Keywords:** Prospective, Headache, Chronic musculoskeletal complaints, Risk factor, Insomnia

## Abstract

**Background:**

A strong relationship between insomnia and painful disorders has been found, but it is still unclear whether chronic pain leads to insomnia. There is a need of large-scale prospective studies to evaluate if there is a causal relationship between painful disorders and insomnia.

**Methods:**

All inhabitants aged ≥ 20 years in Nord-Trøndelag County of Norway were invited to participate in two surveys (n = 92,566 and 93,860, respectively). 27,185 subjects participated in both surveys, and 19,271 of these were insomnia-free at baseline (population at risk). Using logistic regression, we evaluated the influence of headache, CMSCs and coexisting headache and CMSCs on the subsequent risk of insomnia.

**Results:**

Compared to subjects without headache and CMSCs, there was an increased risk of insomnia among those with headache, most pronounced among those with headache ≥ 7 days / month (OR = 2.2, 95% CI = 1.9 – 2.6). Similarly, an increased risk among those with CMSCs was found, most evident for those with widespread CMSCs (OR = 2.0, 95% CI = 1.8 – 2.2). Having coexistent CMSCs and headache (OR = 2.0, 95% CI = 1.8 – 2.2) predisposed more strongly to insomnia than having headache (OR = 1.5, 95% CI = 1.3 – 1.6) and CMSCs (OR = 1.6, 95% CI = 1.4 – 1.7) alone.

**Conclusion:**

In this prospective study headache and CMSCs were risk factors for insomnia 11 years later.

## Background

Insomnia is associated with considerable suffering for the affected individual [1] with additional impact on the society at large [2]. A strong relationship between insomnia and painful disorders have been reported [3], and studies indicate that pain not only might be a risk factor for insomnia but that the two disorders reciprocal influence and exacerbate each other [3]. It is also likely that when insomnia and chronic pain occur together their consequences are even more devastating [4]. In clinical studies acute painful stimuli applied to healthy subjects during sleep resulted in transient arousals [5], while chronic pain patients had poorer sleep than controls in terms of sleep latency, sleep efficiency and awakenings after sleep onset [6].

Although there is a well known association between insomnia and painful disorders it is still unclear whether chronic pain leads to insomnia [3]. The few longitudinal studies that do exist on this topic [7-10] include relatively few participants or lack validated diagnostic criteria of insomnia. The only study that included subjects free of sleep disturbance (n = 437) at baseline, reported that unspecified bodily pain was a risk factor for new-onset insomnia at follow-up one year later [8]. However, to the best of our knowledge, no large follow-up study has evaluated the influence of primary headaches and chronic musculoskeletal complaints (CMSCs) on the risk of insomnia.

In the present study we have studied this topic in a prospective cohort study, using data from two large-scale health surveys carried out in Nord-Trøndelag county of Norway in 1995–1997 and 2006–2008. The main objective was to evaluate the association between primary headaches, CMSCs and coexisting headache and CMSCs at baseline and the subsequent risk of insomnia among men and women at follow-up 11 years later.

## Methods

### The population

Nord-Trøndelag is one of 19 counties in Norway, and its geography and demography is fairly representative for the country [11]. The Nord-Trøndelag Health Survey (“Helseundersøkelsen i Nord-Trøndelag”: HUNT) is a longitudinal cohort study in which all inhabitants ≥20 years old of Nord-Trøndelag have been invited to participate.

### The HUNT 2 study

In HUNT 2 (performed between August 1995 and June 1997) each person completed extensive questionnaires eliciting information on health problems. Among a wide range of topics in the first questionnaire (Q1) were education, physical activity, smoking, alcohol, and anxiety and depression (measured by the Hospital Anxiety and Depression Scale (HADS)) [12]. Details of the phrasing of these questions have been described previously [13-17]. In the present paper, educational level was categorized according to duration (< 13 years vs. ≥ 13 years), work-status as paid work (yes vs. no), and sick leave duration the last 12 months, categorized as 0 weeks, < 2 weeks or ≥ 2 weeks. Cigarette smoking was categorized as current daily smoking (yes vs. no). Reponses to questions on physical activity were categorized according to duration and intensity of exercise per week with the extremes ≥ 3 hours hard physical activity and physical inactivity. Caffeine consumption was subdivided into two groups (highest quartile vs. other quartiles). Alcohol consumption was measured by the CAGE questionnaire [18], using a cutoff ≥ 1 to indicate possible alcohol abuse. Subjects suffering from gastrointestinal (GI) complaints (yes vs. no) were defined as subjects with some or much reflux symptoms and/ or diarrhea and /or constipation and / or nausea. In Q2 participants were asked if they had used medication (for any condition of any type) daily or almost daily during the last 12 months. In the following question they were asked for how many months they had taken specified drugs, including antidepressants, sedatives and hypnotics. We categorized the use of drugs as 0 months vs. ≥ 1 months. They were specifically asked how often, during the last month, they had used sedatives or hypnotics. This question was among those used to define the insomnia-free population in HUNT 2.

### Headache diagnoses

Q2 included 13 questions about headache. All participants were asked the screening question “Have you suffered from headache during the last year?” Subjects answering “yes” were then asked to respond to the other headache-related questions, mainly designed to determine whether subjects met the migraine criteria of the International Headache Society (IHS) [19]. HUNT 2 did not include any question about pain intensity, and criterion C of the migraine diagnosis had to be modified accordingly [11]. Headache not fulfilling the migraine criteria were classified as non-migraineous headache. Headache was defined as frequent if it occurred > 7 days / month. The validity of the questionnaire based headache diagnoses in HUNT 2 has been evaluated previously [11]: For migraine, the sensitivity was 69% and specificity 89% (kappa value 0.59, 95% CI =0.47-0.71); for non-migraineous headache, the sensitivity was 61%, specificity 81% (kappa 0.43, 95% CI=0.29-0.57), and for headache > 7 days/month the sensitivity was 56% and specificity was 91% (kappa 0.50, 95% CI 0.34-0.66).

### Chronic musculoskeletal complaints (CMSCs)

In Q1, subjects were asked the screening question: *“Have you during the last year continuously for at least 3 months had pain and/or stiffness in muscles and joints?”* The reliability of this question has been reported previously [20] and the chance-adjusted agreement (kappa value) was 0.63 (95% CI = 0.53-0.73).

Subjects who answered “yes” were also asked to mark the localization of this pain (neck, shoulders, elbows, wrist/hands, upper back, lower back, hips, knees, and/or ankles/feet). The nine anatomical regions were derived from the Nordic Questionnaire [21] evaluated previously and found to give reliable information for low back, upper limb and neck on the presence of symptoms during the past year [22].

Subjects who answered “yes” to the screening question were considered to have CMSCs. Widespread CMSCs were defined according to the 1990 American College of Rheumatology (ARC) as CMSCs from all of the following three regions: axial skeleton (neck, upper back, or lower back), above the waist (neck, shoulders, elbows, wrist/hands, or upper back) and below the waist (lower back, hips, knees, or ankles/feet) [23]. In HUNT 2 participants were not asked to distinguish between pain in the left and right side of the body. The change-adjusted agreement (kappa value) for widespread CMSCs has recently been estimated to 0.48 (95% CI = 0.38-0.64) [20]. Subjects with CMSCs who did not meet the criteria for widespread CMSCs were defined as non-widespread CMSCs.

### The definition of insomnia-free in HUNT 2

In Q2, subjects were asked two questions about the frequency of sleep onset insomnia (“Have you had problems falling to sleep in the last month?”) and sleep maintenance problems (“During the last month, did you ever wake up too early, not being able to fall asleep again?”). There were four possible responses: 0 = never, 1 = occasionally, 2 = often, or 3 = almost every night. These two insomnia symptoms were dichotomized into: 0 = “no” (never / occasionally) or 1 = “yes” (often / almost every night). These two answer scores were added, and subjects with a sum score of 0 were considered unlikely to have insomnia [24-26]. In the present study insomnia-free was defined as those who had a sum score of 0 on the two insomnia questions *and* had never used hypnotics or **s**edatives the last month.

### The HUNT 3 study

HUNT 3 (performed between October 2006 and June 2008) was to a large extent a replication of HUNT 2.

### Insomnia diagnosis

HUNT 3 included three insomnia questions in Q2: “How often during the *last three months* have you experienced: 1) difficulty falling asleep at night, 2) several awakenings during the night and 3) wake up to early and were not able to fall asleep again. The response alternatives were: 0 = never or seldom, 1 = sometimes, 2 = several times a week. The reliability of these three insomnia questions in HUNT 3 has been assessed previously, and found to be moderate with kappa-values of 0.44 (95% CI = 0.35 – 0.52) and 0.38 (95% CI = 0.31 – 0.46) and 0.35 (95% CI = 0.26 – 0.46) respectively [27]. To create a DSM-IV based proxy insomnia [24] diagnosis the three questions were first dichotomized into two possible responses: 0 = “no” (never, seldom or sometimes) and 1 = “yes” (several times a week). The insomnia questions were then added, and subjects with a score ≥ 1 were considered likely to have insomnia.

### Headache

The Q2 also included the same headache-screening question that was used in HUNT 2. For the questionnaire-based status as a headache sufferer, a sensitivity of 88%, a specificity of 86%, and a kappa value of 0.70 have been found [28].

#### Study population

In HUNT 2, 65, 237 (69.5%) of 92,566 invited individuals answered Q1**,** and 50,738 (54.8%) answered the insomnia questions in Q2 and were classifiable according to pain status. Details of non-responders have been described previously [29]. In HUNT 3, 50,807 (54.1%) of the 93,860 invited adults answered Q1, and 40,534 (43.2%) completed the insomnia questions in Q2.

**Figure 1:**
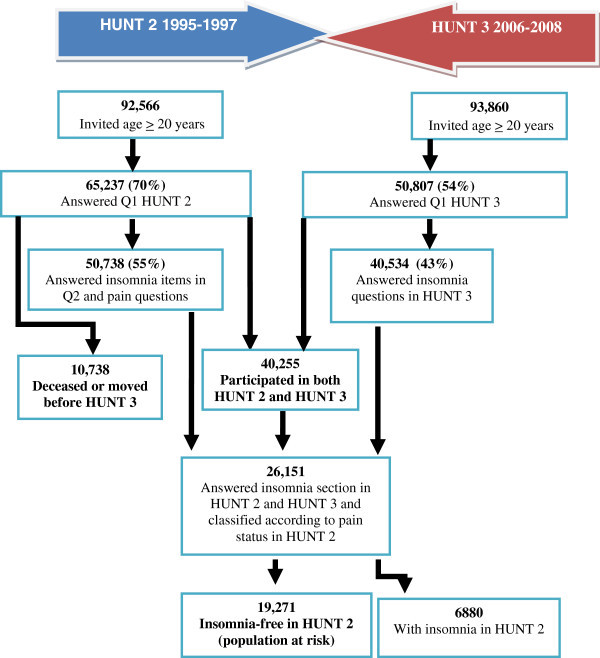
The flow of participants in HUNT 2 and HUNT 3.

Among the 65,237 participants in HUNT 2, 10,507 had died, 240 had emigrated and 1 had disappeared by the time of HUNT 3. Out of all the 54,789 eligible HUNT 2 participants, 69% of men and 72% of women also participated in HUNT 3. The mean time of follow-up between HUNT 2 and HUNT 3 was 11 (range 9–13) years. Among the 50,738 who answered the insomnia in HUNT 2, 26,151 answered the insomnia related questions in both surveys. Of these 19,271 (74%) were considered free of insomnia in HUNT 2 (sum score of 0 on the proxy insomnia diagnosis and did not report any use of sleep medication or sedatives) and were defined as the population at risk. The remaining 6,880 had insomnia-symptoms at baseline in HUNT 2. The flow chart of participants in HUNT 2 and HUNT 3 is shown in Figure 1.

### Ethics

The study was approved by the Regional Committee for Ethics in Medical Research.

### Statistics

In the population at risk, we used multivariate logistic regression (with 95% confidence intervals [CI]) to evaluate the relative influence of headache, CMSCs (alone or in combination with each other) in HUNT 2 on the risk of insomnia at follow-up. Comparisons were done between men and women to detect possible gender differences. To evaluate the probability of a linear relationship between headache frequency at baseline and insomnia at follow up, we performed trend analyses by treating frequency of headache (three categories) as a single ordinal variable. The trend test was considered statistically significant at p < 0.05.

In the multivariate analyses we initially adjusted for age and gender. In a second set of analyses additional adjustments were done for previously identified potential confounding factors [2,10,13-16,29], in particular education level, work-status, sick leave, level of physical activity, daily use of tobacco, alcohol overuse, high coffee-consumption, anxiety and depression, antidepressants, GI-complaints, body mass index (BMI) and systolic blood pressure (BP). The variables were categorized as described in the method section (age, BMI and systolic BP as continuous variables), and included in the statistical model one at a time. Potential confounders were excluded in the final analyses if the OR changed less than 0.1 in analyses regarding headache and CMSCs respectively. Subjects with incomplete data regarding one or several variables were included in the analysis (as a “missing category”) to reduce the impact of possible bias.

In supplementary adjusted analyses, we evaluated the influence of headache status in HUNT 2 and HUNT 3 on the odds of having insomnia in HUNT 3.

The statistical analyses were performed with the Predictive Analytics Software (PASW), Statistics version 17.0 by Inc., an IBM Company (Chicago, IL, USA).

## Results

Demographic and clinical characteristics of the population at risk (n=19,271) are given in Table 1.

**Table 1:** **Baseline characteristics of the population at risk**^**1 **^**in HUNT-2**

**Variables**	
Number of participants	19,271
Age (mean [SD], missing = 0)	45.8 [12.7]
Gender (% women, missing = 0)	54.7
Married (%, missing = 12)	69.4
Education ≥ 13 years (%, missing = 275)	35.6
Paid work (%, missing = 167)	73.6
High level of physical activity^2 ^(%, missing = 4150)	9.7
BMI (mean [SD], missing = 35)	26.0 [3.8]
Systolic BP (mean [SD], missing = 46)	133.3 [18.4]
Daily use of tobacco (%, missing = 1857)	27.2
Alcohol overuse (cage score ≥ 1) (%, missing = 2287)	16.2
Total HADS-score (mean [SD], missing = 1978)	6.6 [4.7]
GI-complaints^3^ (%, missing 517)	48.5

^1^: Population at risk = Insomnia-free subjects (defined as subjects who have never or occasionally had sleep onset or terminal insomnia during the last month, and do not use sleep medication) in HUNT-2 who answered the questions about headache and musculoskeletal complaints.

^2^: High level of physical activity = Strenuous physical activity ≥ 3 hours / week.

^3^: GI-complaints: Some or much reflux symptoms and/ or diarrhea and /or constipation and / or nausea.

### Headache

In the multivariate analysis, adjusting for age and sex, GI-complaints and total HADS score, headache at baseline was associated with a 2-fold risk of insomnia (OR = 1.7, 95% CI = 1.6 – 1.9) compared to subjects without headache and CMSCs. Compared to subjects without headache and CMSCs those with headache < 7 days / month had a 70% increased risk, while those with headache ≥ 7 days had a 120% increased for insomnia (Table 2). There was a significant relationship (p trend <0.001) between the headache frequency at baseline and the risk of insomnia 11 years later (Table 2). There were no significant differences between headache types or gender (Table 2).

**Table 2:** Influence of headache (related to type and frequency) on the risk of insomnia in HUNT-3 among the population at risk (n = 19,271)

			**Both gender**^**1**^		**Women**^**2**^		**Men**^**2**^
			**Insomnia**^**3**^		**Insomnia**^**3**^		**Insomnia**^**3**^
	n, total = 14,639	n = 3069	OR (95% CI)	n = 2020	OR (95% CI)	n = 1049	OR (95% CI)
**No headache or CMSCs**	**7263**	**1080**	**1.0 (ref)**	**581**	**1.0 (ref)**	**499**	**1.0 (ref)**
Headache^4^	7376	1989	1.7 (1.6 – 1.9)	1439	1.8 (1.6 – 2.0)	550	1.7 (1.4 – 1.9)
< 7 days / month	6202	1589	1.7 (1.5 – 1.8)	1142	1.7 (1.5 – 1.9)	447	1.6 (1.4 – 1.8)
≥ 7 days / month	1174	400	2.2 (1.9 – 2.6)	297	2.2 (1.8 – 2.7)	103	2.2 (1.7 – 2.8)
p-trend			<0.001		<0.001		<0.001
Non migraineous headache	4963	1278	1.7 (1.5 – 1.8)	884	1.7 (1.5 – 1.9)	394	1.6 (1.4 – 1.9)
< 7 days / month	4279	1049	1.6 (1.4 – 1.8)	726	1.6 (1.4 – 1.8)	323	1.6 (1.3 – 1.8)
≥ 7 days / month	684	229	2.2 (1.8 – 2.7)	158	2.2 (1.7 – 2.8)	71	2.3 (1.6 – 3.1)
p-trend			<0.001		<0.001		<0.001
Migraine	2413	711	1.9 (1.7 – 2.1)	555	2.0 (1.7 – 2.3)	156	1.7 (1.4 – 2.1)
< 7 days / month	1923	540	1.8 (1.6 – 2.1)	416	1.9 (1.6 – 2.2)	124	1.6 (1.3 – 2.1)
≥ 7 days / month	490	171	2.2 (1.7 – 2.7)	139	2.2 (1.7 – 2.9)	32	2.0 (1.3 – 3.1)
p –trend			<0.001		<0.001		<0.001

^1^Data is adjusted for age, gender, anxiety, depression (total HADS-score) and GI-complaints.

^2^Data is adjusted for age, anxiety, depression (total HADS-score) and GI-complaints.

^3^: Insomnia = During the last three months difficulty falling asleep at night and / or several awakenings during the night and / or wake up to early and were not able to fall asleep again several days a week.

^4^: Headache = Subjects with headache only or headache and CMSCs combined.

Missing data: 155 subjects with pure headache (= 4.2%) and 182 subjects with coexisting headache and CMSCs (= 4.5%) did not answer the question on headache frequency.

### Chronic musculoskeletal complaints

In age-, sex-, GI-complaints and total HADS score adjusted analyses, CMSCs at baseline was associated with a 2-fold risk of insomnia at follow-up (OR = 1.8; 95% CI = 1.6 – 1.9) compared to subjects without headache and CMSCs. Compared to subjects without headache and CMSCs non-widespread CMSCs gave a 60% increased risk of insomnia, while a 100% increased risk was observed among those with widespread CMSCs (Table 3). There were no significant gender differences (Table 3).

**Table 3:** Influence of CMSC on the risk of insomnia in HUNT-3 among the population at risk (n = 19,271)

			**Both gender**^**1**^		**Women**^**2**^		**Men**^**2**^
			**Insomnia**^**3**^		**Insomnia**^**3**^		**Insomnia**^**3**^
	n, total = 15,604	n = 3372	OR (95% CI)	n = 2025	OR (95% CI)	n = 1347	
**No headache or CMSCs**	**7263**	**1080**	**1.0 (ref)**	**581**	**1.0 (ref)**	**499**	**1.0 (ref)**
CMSCs^4^	8341	2292	1.8 (1.6 – 1.9)	1444	1.9 (1.6 – 2.1)	848	1.7 (1.5 – 1.9)
Non widespread CMSC	4219	992	1.6 (1.4 – 1.7)	584	1.6 (1.4 – 1.9)	408	1.5 (1.3 – 1.8)
Widespread^5^ CMSC	4122	1300	2.0 (1.8 – 2.2)	860	2.1 (1.8 – 2.4)	440	1.9 (1.6 – 2.2)

^1^Data is adjusted for age, gender, anxiety, depression (total HADS-score) and GI-complaints.

^2^Data is adjusted for age, anxiety, depression (total HADS-score) and GI-complaints.

^3^: Insomnia = During the last three months difficulty falling asleep at night and / or several awakenings during the night and / or wake up to early and were not able to fall asleep again several days a week.

^4,1^: CMSCs is defined as the subjects who answered “yes” to the screening question: “Have you during the last year continuously for at least 3 months had pain and / or stiffness in muscles and joints?”

^4,2^: CMSCs = Subjects with CMSCs only or headache and CMSCs combined.

^5^: Widespread CMSCs = CMSCs from all of the following three regions: axial skeleton (neck, upper back, or lower back), above the waist (neck, shoulders, elbows, wrist/hands, or upper back) and below the waist (lower back, hips, knees, or ankles/feet).

### Combined headache and chronic musculoskeletal complaints

Compared to subjects without headache and CMSCs, pure headache < 7 days / month at baseline gave a 40% increased risk of insomnia, while pure headache ≥ 7 days / month gave a 110% increased risk (Table 4). The combination of headache and CMSCs predisposed more strongly to insomnia than headache alone (Table 4). This effect was most prominent and significant (non-overlapping CIs) for headache <7 days / month with a 90% increased risk of insomnia compared to subjects free of headache and CMSCs at baseline (Table 4). Regarding subjects with headache ≥ 7 days / month having CMSCs did not have any strong influence the risk for insomnia (OR = 2.2, 95% CI = 1.9 – 2.7), compared to headache alone (overlapping CIs in Table 4).

**Table 4:** **Risk of insomnia**^**1 **^**at 11-year follow-up according to the combined effect of headache (related to type and frequency) and CMSCs**^**2 **^**(yes/no)**

		**Pure headache (no CMSCs)**			**Coexisting headache and CMSCs**	
		**Insomnia**^**1**^			**Insomnia**^**1**^	
	total, n = 10,775	n = 1896	OR (95% CI)	total, n = 11,127	n = 2283	OR (95% CI)
**No headache or CMSCs**	**7263**	**1080**	**1.0 (ref)**	**7263**	**1080**	**1.0 (ref)**
Headache	3512	786	1.5 (1.3 – 1.6)	3864	1203	2.0 (1.8 – 2.2)*
< 7 days / month	3141	669	1.4 (1.2 – 1.6)	3061	920	1.9 (1.7 – 2.2)*
≥ 7 days / month	371	117	2.1 (1.7 – 2.7)	803	283	2.2 (1.9 – 2.7)
p-trend			<0.001			<0.001
Non migraineous headache	2437	512	1.4 (1.2 – 1.5)	2526	766	2.0 (1.7 – 2.2)*
< 7 days / month	2221	443	1.3 (1.1 – 1.5)	2058	606	1.9 (1.6 – 2.2)*
≥ 7 days / month	216	69	2.2 (1.6 – 3.0)	468	160	2.2 (1.8 – 2.8)
p-trend			<0.001			<0.001
Migraine	1075	274	1.7 (1.5 – 2.0)	1338	437	2.0 (1.7 – 2.3)
< 7 days / month	920	226	1.7 (1.4 – 2.0)	1003	314	1.9 (1.7 – 2.3)
≥ 7 days / month	155	48	2.1 (1.5 – 3.0)	335	123	2.2 (1.7 – 2.8)
p –trend			<0.001			<0.001

*Significant differences (non-overlapping CIs).

All data are adjusted for age, gender, anxiety, depression (total HADS-score) and GI-complaints.

^1^: Insomnia = During the last three months difficulty falling asleep at night and / or several awakenings during the night and / or wake up to early and were not able to fall asleep again several days a week.

^2^: CMSCs = Answered “yes” to the screening question: “Have you during the last year continuously for at least 3 months had pain and / or stiffness in muscles and joints?”

Missing data: 155 subjects with pure headache (= 4.2%) and 182 subjects with coexisting headache and CMSCs (= 4.5%) did not answer the question on headache frequency.

As shown in Table 5, compared to subjects without headache and CMSCs, the coexistence of headache ≥ 7 days / month and CMSCs had a stronger (significant) influence on the risk of insomnia (overall OR = 2.2, 95% CI = 1.9 – 2.6), than pure CMSCs (overall OR = 1.6, 95% CI = 1.4 – 1.7). This effect was evident for both non-widespread CMSCs and widespread CMSCs, but only significant for the former (Table 5).

**Table 5:** **Risk of insomnia**^**1 **^**at 11-year follow-up according to the combined effect of CMSCs**^**2 **^**(non-widespread vs. widespread) and headache (yes/no)**

		**Pure CMSCs (no headache)**			**CMSCs & headache < 7d / month**			**CMSCs & headache ≥7d / month**	
		**Insomnia**^**1**^			**Insomnia**^**1**^			**Insomnia**^**1**^	
	N, total = 11,558	N, total = 2109	OR (95% CI)	N, total = 10,324	N, total = 2000	OR (95% CI)	N, total = 8066		OR (95% CI)
**No headache or CMSCs**	**7263**	**1080**	**1.0 (ref)**	**7263**	**1080**	**1.0 (ref)**	**7263**	**1080**	**1.0 (ref)**
CMSCs^2^	4295	1029	1.6 (1.4 – 1.7)	3061	920	1.9 (1.7 – 2.2)	803	283	2.2 (1.9 – 2.6)*
Non-widespread CMSCs	2513	540	1.4 (1.3 – 1.6)	1357	339	1.6 (1.4 – 1.9)	262	86	2.2 (1.7 – 3.0)*
Widespread^3^ CMSCs	1782	489	1.8 (1.5 – 2.0)	1704	581	2.2 (1.9 – 2.5)	541	197	2.2 (1.8 – 2.7)

*Significant differences compared to pure CMSCs (no headache) (non-overlapping CIs).

All data are adjusted for age, gender, anxiety, depression (total HADS-score) and GI-complaints.

^1^: Insomnia = During the last three months difficulty falling asleep at night and / or several awakenings during the night and / or wake up to early and were not able to fall asleep again several days a week.

^2^: CMSCs = Have answered “yes” to the screening question: “Have you during the last year continuously for at least 3 months had pain and / or stiffness in muscles and joints?”

^3^: Widespread CMSCs = CMSCs from all of the following three regions: axial skeleton (neck, upper back, or lower back), above the waist (neck, shoulders, elbows, wrist/hands, or upper back) and below the waist (lower back, hips, knees, or ankles/feet).

Missing data: 155 subjects with pure headache (= 4.2%) and 182 subjects with coexisting headache and CMSCs (= 4.5%) did not answer the question on headache frequency.

### Influence of headache status in HUNT 2 and HUNT 3

Individuals with headache in both surveys were more strongly associated to insomnia in HUNT 3 (OR=2.0; 95% CI 1.8-2.2) than those with headache in HUNT 3 only (OR=1.7, 95% CI 1.5-1.9) and those with headache in HUNT 2 only (OR=1.3, 95% CI 1.1-1.4), using headache-free subjects in both surveys as a reference group.

## Discussion

In this large-scale prospective study we found that headache and CMSCs increased the risk of insomnia at follow-up, most evident among those with headache ≥ 7 days/month, widespread CMSCs and the co-occurrence of headache and CMSCs at baseline.

Strengths of the present study include a well-defined population at risk without insomnia and the longitudinal design. In the large and unselected population we had data allowing separate analyses to evaluate the impact of pure headache as well as the coexistence of headache and CMSCs at baseline on the risk of insomnia. Furthermore, we had previously validated the questionnaire-based headache diagnoses [11], which have sensitivity estimates varying between 56% and 69% and a high specificity varying between 89% and 91%. We also found that the screening question for CMSCs had good reliability [20]. In addition, the present study was relatively well powered to detect gender differences. To the best of our knowledge, no previous study has included such a large number of other relevant health-related variables. This made it possible to adjust for several potential confounding variables including anxiety and depression [30]. Importantly, although we adjusted for anxiety and depression in the present paper, this does not rule out the possibility that these disorders are likely to play a major role in the development of insomnia in subjects with chronic painful conditions [3,4]. Also, the possibility of residual confounding by unrecognized factors cannot be ruled out.

Some limitations should be considered. First, similar to other relevant prospective studies, the assessment of insomnia was questionnaire-based. Insomnia may, however, be present even in the absence of any objective measures for example by polysomnography [31]. Second, although the present proxy-insomnia diagnosis has been used successfully in several other studies [24,25], it has not yet been validated and it does not fulfill the DSM-IV criteria for insomnia completely. Both surveys lacked sufficient information about daytime impairment that is required in the International Classification of Disease, ninth (ICD-9) and tenth (ICD-10) edition and the DSM-IV criteria. As described in the method-section, the HUNT 3 study did include one question regarding daytime sleepiness. However, there are conflicting results regarding the association between insomnia and subjective daytime sleepiness [32]. Further, as the daytime consequences vary between different insomnia subtypes [33], and even from person to person [34], it is unlikely that one question could capture all individuals with insomnia causing daytime impairment [34]. The insomnia diagnosis in HUNT 2 is also different from the DSM-IV criteria in another aspect, as the questionnaire did not include information about the insomnia symptoms beyond one month. While the ICD-9 and ICD-10 criteria have no requirement on the duration of the insomnia symptoms, it has to be present for more than one month according to the second version of the International Classification of Sleep Disorders (ICSD-2) [35] and the DSM-IV [36]. This makes our insomnia criteria less specific than the DSM-IV criteria, which might have influenced the results of the present study. In effort to make the diagnoses more specific we excluded subjects using hypnotics or sleep medication from the population at risk in HUNT 2, and based the diagnosis in HUNT 3 on three insomnia symptoms instead of two. Ideally the diagnoses would have been identical in HUNT 2 and HUNT 3, but unfortunately the insomnia questions in the two surveys differed a bit.

Third, although 62% of the subjects who answered the insomnia section of HUNT 2 also answered the insomnia section in HUNT 3, a selection bias cannot be ruled out. The participation rate was moderate at baseline (58%) and only 29% of the total population completed the insomnia section in both surveys. Despite this limitation, the HUNT studies include more than 200 health related variables, which will probably ensure that any remaining bias relevant to insomnia, headache and CMSCs is negligible.

Few previous longitudinal studies have evaluated the influence of headache and CMSCs on the risk of insomnia, and no large population-based follow-up study of adults has similar objectives and study design. In line with the present results, there is one study of 437 participants [8] that reported that unspecified bodily pain was a risk factor for new-onset insomnia at follow-up 1 year later. Furthermore, longitudinal studies have found rheumatoid arthritis [9], painful musculoskeletal conditions [7] and migraine [10] to have a negative influence on pre-existing sleep difficulties.

In the latter study by Singareddy et al. [10] migraine was identified as a significant risk factor for insomnia in the univariate analyses, but not in the multivariate analyses. However, in their multivariate analyses migraine was included in a ”mixed group of individuals with physical health complaints” consisting of e.g. asthma, allergy, anemia, and kidney disease. The study by Singareddy et al. also included other important methodological differences which may explain divergent results, like different population at risk (they included subjects with poor sleep), different way to diagnose migraine (self rapport according to ’physical based diagnosis’), and number of study participants (1246 versus 19,271 in the present study).

The present study provides evidence that headache (migraine and non-migraineous) and CMSCs increases the risk of insomnia. Individuals with persistent headache in both surveys had the strongest association with insomnia in HUNT 3. The fact that both headache and CMSCs increased the risk of insomnia might reflect that the found association is not attributed to either of the conditions themselves, but more generally linked to the experience of pain over time. Indeed, several other painful conditions are linked to poor sleep [7-9]. Further, the results of the present study were most evident for headache ≥ 7 days / month, in accordance with other studies emphasizing the importance of the frequency of pain [37]. Interestingly, we have previously identified insomnia as a risk factor for primary headaches 11 years later [26]. Thus, as previous suggested by other studies [38] there might be a reciprocal relationship between headache and insomnia, with each disorder increasing the risk of onset of the other. In contrast, in a study on a mixed group of pain disorders (headache included), pain was not a reliable predictor for subsequent sleep, while cognitive arousals could predict the ensuing sleep quality [39]. In the same study, sleep quality was a predictor of pain the next day, and good sleep quality relieved pain the first half of the day [39]. However, this study looked at the short-time relationship between pain and sleep [39], which might differ from a long term perspective.

If a reciprocal relationship between headache and insomnia do exist, they can be causally related, with insomnia causing headache, and vice versa, but one cannot exclude the possibility of a common neurobiological substrate predisposing to both conditions [38]. Although the exact nature of this bidirectional relationship remains unclear, it might lead to an evil circle where one disorder exacerbates the other [3]. The trigger might be either of the conditions [40], and regarding the results of the present study there are several theories on how pain might lead to sleep difficulties.

One possible mechanism is that chronic pain leads to structural changes in areas of the brain regulating sleep [40]. Pain may also cause cognitive pre-sleep arousal, which disturbs the initiation of sleep [37]. Others believe that painful conditions like headache might lead to sleep-seeking behavior to relieve the pain, which in turn leads to bad sleep habits and insomnia [41].

One interesting aspect of these theories is that better pain management might relieve sleep difficulties as well [3]. If there is a bidirectional relationship between painful conditions and sleep disturbances, patients might benefit from a more systematic approach to treatment of insomnia, as well as headache and CMSCs [3]. Further prospective studies should evaluate whether effective treatment of headache and CMSCs could prevent future insomnia.

## Conclusion

Headache and CMSCs (particularly frequent headache, widespread CMSCs or the combination of the two) increase the risk for insomnia after 11 years.

## Abbreviations

ARC: American College of Rheumatology; BMI: Body Mass Index; BP: Blood pressure; CAGE: Cut down, Annoyed, Guilty and Eye-opener (screening tool for alcohol dependency); CI: Confidence Interval; CMSCs: Chronic musculoskeletal complaints; DSM-IV: The Diagnostic and Statistical Manual for Mental Disorders, Fourth Edition; GI: Gastrointestinal; HADS: The Hospital Anxiety and Depression Scale; HUNT: The Nord-Trøndelag Health Study; ICD-9: International Classification of Disease, ninth Edition; ICD-10: International Classification of Disease, tenth Edition; ICSD-2: The International Classification of Sleep Disorders, second Edition; IHS: International Headache Society; OR: Odds ratio.

## Competing interests

The authors declare that they have no competing interests.

## Author’s contributions

All authors participated in the design of the study. SSØ were responsible for the statistical analysis and interpretation of data, and drafted the manuscript with guidance of KH. TS, ME, JAZ and KH contributed with critical revision of the manuscript for important intellectual content. All authors read and approved the final manuscript.
